# Epidemiology of civilian patients with acute conflict-related extremity injuries sustained in Syria and Iraq

**DOI:** 10.1371/journal.pgph.0006627

**Published:** 2026-07-10

**Authors:** Dennis Bengtson, Rawand Haweizy, Khaldoon Bashaireh, Jonas Malmstedt, Andreas Älgå

**Affiliations:** 1 Department of Global Public Health, Karolinska Institutet, Stockholm, Sweden; 2 College of Medicine, Hawler Medical University, Erbil, Iraq; 3 Department of Special Surgery, Jordan University of Science and Technology, Irbid, Jordan; 4 Department of Clinical Science and Education, Södersjukhuset, Karolinska Institutet, Stockholm, Sweden; Muhlenberg College, UNITED STATES OF AMERICA

## Abstract

Most casualties of modern conflict are civilian, and extremity injuries are the most frequent injury type. However, prospectively epidemiological data on civilians with conflict-related extremity injuries remain scarce. We conducted a multi-site observational study of prospectively collected data from a randomized controlled trial on negative pressure wound therapy. The present study disregarded treatment modality and described adults with acute conflict-related extremity wounds not suitable for primary closure treated at two civilian trauma hospitals in Erbil, Iraq and Ar Ramtha, Jordan from 2015 until 2018. A total of 165 patients were included. Median age was 28 years (interquartile range [IQR] 21–34); 155 (94%) were male. Gunshot injuries were most common (100 [60%] patients), followed by blast injuries (63 [38%] patients). Fractures occurred in 93 (57%) patients, including 74 (45%) open fractures, of which 16 (22%) had wound infections, defined as purulent discharge. Concomitant injuries occurred in 43 (26%) patients, including fractures at a site other than the studied wound, penetrating abdominal injuries, and abdominal solid-organ or gastrointestinal injuries. Bleeding requiring transfusion affected 56 (35%) patients. Median hospital stay was 10 days (IQR 5–37), and one (1%) patient died. Patients treated in Ar Ramtha had more wounds, more concomitant injuries, more blast injuries, longer times to wound closure, and longer hospital stays than patients treated in Erbil. Fractures were common, including open fractures, and bleeding requiring transfusion was frequent. Infection among patients with open fractures was comparatively uncommon, although comparison with other settings is limited by differences in case mix and organization of care. Low in-hospital mortality likely reflects selection of patients who survived to reach hospital. By providing standardized prospective data on an underreported civilian conflict-injury population, this study may inform public health planning for surgical capacity, blood supply, wound care, and hospital resources in similar conflict-affected settings.

**Trial registration:**

NCT02444598

## Introduction

In June 2023 – June 2024, the global incidence of conflict-related fatalities amounted to 216 thousand deaths [1]. The total number of deaths from armed conflict as compared to the same period 2020 – 2021 had increased by 44%, largely attributable to the conflict in Tigray, and the ongoing conflicts in Ukraine and Palestine [1]. As per the most recent Global Burden of Disease (GBD) study, the total conflict-related injuries caused the loss of 8.73 million disability-adjusted life years in 2021, a number likely to have increased in conjunction with the increase in global fatalities [2].

Conflict-related death and injury predominantly affect civilians [3,4]. The most common site of conflict-related injury among civilians are the extremities [4–6]. Acute challenges of conflict-related extremity injuries among civilians include bleeding, infection, and traumatic amputation [7]. In the long-term, extremity injuries from conflict lead to psychological distress, lost quality of life, and poverty [8,9].

The current gold standard treatment of conflict-related extremity injuries includes initial debridement, applying a dressing to protect the wound from further contamination until reassessment, and, if possible, delayed wound closure in the operating theatre 3–5 days after initial surgery [10,11]. Prophylactic antibiotics are recommended as an adjunct to adequate surgical treatment [10]. Primary closure is not advisable except in rare circumstances, such as a low-energy mechanism of injury and with a clean, recent, and non-contaminated wound [10].

Prospective epidemiological data on civilians with conflict-related extremity injuries remain scarce [6]. In conflict settings, systematic data collection is hampered by damaged health information systems and telecommunications, threats to healthcare professionals, forced population movement, and fragmented records [12–16]. These conditions increase the risk that retrospective studies are affected by missing data, recall bias, and misclassification [17]. The present study uses prospectively collected data from a randomized controlled trial with predefined eligibility criteria, standardized data collection, and comparable measurements across two civilian trauma hospitals.

### Aim

By utilizing prospectively collected data we aim to increase the understanding of the epidemiology of patients with acute conflict-related extremity injuries not suitable for primary closure treated at civilian hospitals.

## Methods

### Ethics statement

This analysis was part of a clinical trial which received ethical approval from the Ethics Review Committee of the Jordan Ministry of Health (MOH REC 150037), the Ethics Review Board of Médecins Sans Frontières (ID 1520), the Research Ethics Committee, Kurdistan Regional Government in Iraq (2:10 6/3/2017), and the Swedish Ethical Review Authority (2019–01975). All participants provided written informed consent.

### Study design

We conducted an analysis of prospective clinical data derived from a pragmatic randomized controlled trial on the effectiveness of negative pressure wound therapy (NPWT) among civilians with conflict-related injuries [18]. The trial found that NPWT was not superior to standard wound treatment [19]. The present study focuses on the epidemiological characteristics of the participants of the trial as a whole, disregarding treatment modality and stratifying patients by study site.

### Study setting

Data were collected at two civilian trauma hospitals located in Erbil, Iraq and Ar Ramtha, Jordan. The hospital in Erbil predominately received patients with conflict-related injuries from Mosul, Iraq and is run by a local non-governmental organization, Emergency Management Center. The hospital is situated 85 kilometers from the center of Mosul. The hospital in Ar Ramtha treated patients having sustained conflict-related injuries in Syria and was supported by the international non-governmental organization Médecins Sans Frontières. The distance between the hospital and the Syrian-Jordan border is 6 km. Both hospitals were supplied with blood products via regional blood banks.

### Participants

To be included in the trial from which our data were derived, the patient had to be an adult (≥18 years) with at least one conflict-related extremity wound not suitable for primary closure presenting within 72 hours after the time of injury [18]. Patients were recruited from June 9^th^, 2015, until 5^th^ February 2017 at the hospital in Ar Ramtha, and from April 20^th^ 2017 until October 24^th^, 2018 at the hospital in Erbil. As neither hospital offered neurosurgical services nor services for severe burn victims, patients with central nervous system (CNS) injuries or severe burns were excluded and referred to other hospitals. Patients injured in a mass casualty event or who underwent primary surgery at a time when a research nurse was unavailable were not included. A total of 165 patients were included.

### Data analysis

Continuous variables were reported as medians with interquartile ranges (IQR) and binary variables as counts and percentages. Mann-Whitney U test was used for group comparisons for continuous variables, whilst Fisher’s exact test was used to test binary variables. Effect sizes were reported as the Hodges-Lehmann estimator (continuous variables) [20], or as odds ratios (binary variables). Odds ratios were not calculated for binary variables with zero-cells.

Bleeding requiring transfusion was based on local guidelines and the attending surgeon’s judgement. Wound infection was operationalized as purulent discharge from the wound. Any concomitant injury was defined as a fracture at a different site than the randomized wound, a urogenital injury, an injury of an abdominal solid organ or the GI tract, a lung injury, a penetrating injury to the thorax or trachea, or a penetrating injury to the abdomen.

Missing data were handled using pairwise deletion. When applicable, percentages were calculated on the number of patients not missing data. Sensitivity to missing data was tested by median value imputation for continuous variables, and by best/worse-case analysis for binary variables (i.e., by assigning all patients missing for one site to an event and all missing from the other to a non-event, and then conducting the same test in reverse direction). No p-values changed significance during sensitivity analysis.

All numbers were rounded to whole numbers, except for hemoglobin values, which were rounded to the nearest decimal. Age and sex distribution as well as blood, platelet, and plasma administration were analyzed using intervals of values. Statistical processing was done using R version 4.2.1, (R Core Team, 2022).

### Inclusivity in global research

Additional information regarding the ethical, cultural, and scientific considerations specific to inclusivity in global research is included in the Supporting Information (S1 Checklist).

## Results

The patients were predominantly young males with a median age of 28 years (IQR 21–34) (Table 1). Most patients (106 [64%]) were smokers, and 13 (8%) patients had comorbidities. The typical patient presented with more than one wound and had stable vital parameters on admission. Concomitant injuries were common, with 43 (26%) patients suffering at least one concomitant injury. Fractures were present for most patients, with 93 (57%) patients having at least one fracture. Some 100 (60%) patients were injured by gunshot and 63 (38%) patients had blast injuries. Compared with patients arriving to Ar Ramtha, patients arriving to Erbil typically had fewer wounds (2 vs 4, 95% CI for the difference: 1–3), were less likely to have concomitant injuries (6% vs 55%, OR 0.05, 95% CI 0.02–0.14), and were less likely to have a fracture (47% vs 72%, OR 0.35, 95% CI 0.18–0.71). Compared with patients arriving to Ar Ramtha, patients arriving to Erbil were more likely to have sustained gunshot injuries (85% vs 25%, OR 15.91, 95% CI 7.21–35.4) and less likely to have sustained blast injuries (14% vs 73%, OR 0.06, 95% CI 0.03–0.14). Surgery before admission was less common among patients arriving to Erbil than among those arriving to Ar Ramtha (OR 0.16, 95% CI 0.04–0.6).

**Table 1:** Clinical characteristics (n = 165).

Variable	Total (n = 165)	Erbil (n = 98)	Ar Ramtha (n = 67)	Effect size (95% CI)	P-value
Age, years	28 (21–34)^1^	29 (23–35)^1^	26 (20–33)	1 (-1–4)	0.35
Sex, male	155 (94%)	90 (92%)	65 (97%)	0.35 (0.05–1.61)	0.20
Currently smoking	106 (64%)	65 (66%)	41 (61%)	1.25 (0.65–2.38	0.52
Other disease(s)	13 (8%)	7 (7%)	6 (9%)	0.78 (0.24–2.5)	0.77
Time from injury to admission <48 hours	163 (99%)	96 (98%)	67 (100%)	-^a^	0.51
Surgery before admission	14 (8%)	3 (3%)	11 (16%)	0.16 (0.04–0.6)	**<0.005**
Pulse on admission, beats/minute	100 (85–110)^2^	93 (86–104)^3^	100 (85–113)^4^	-3 (-10–3)	0.35
Systolic blood pressure on admission, mm Hg	120 (110–130)^5^	120 (110–130)^6^	122 (111–136)^1^	-2 (-10–2)	0.24
Hemoglobin level on admission, grams/liter	13.3 (11.3–14.4)^7^	14.1 (12.4–14.9)^8^	12 (10.3–13.6)^1^	1.9 (1.2–2.6)	**<0.005**
Number of wounds	2 (2–4)	2 (2–2)	4 (2–6)	-2 (-3–-1)	**<0.005**
Penetrating injury to the abdomen	10 (6%)	0 (0%)	10 (15%)	-^a^	**<0.005**
Injury of abdominal solid organ or GI tract	9 (5%)	0 (0%)	7 (10%)	-^a^	**<0.005**
Penetrating injury to the thorax or trachea	6 (4%)	1 (1%)	5 (7%)	0.13 (0.01–0.98)	**0.041**
Urogenital injury	6 (4%)	1 (1%)	5 (7%)	0.13 (0.01–0.98)	**0.041**
Lung injury	1 (1%)	0 (0%)	1 (1%)	-^a^	0.41
Fracture at site other than studied wound	33 (20%)^1^	6 (6%)^1^	27 (40%)	0.10 (0.04–0.26)	**<0.005**
Any concomitant injury	43 (26%)	6 (6%)	37 (55%)	0.05 (0.02–0.14)	**<0.005**
Fracture at studied wound	74 (45%)^1^	40 (41%)^1^	34 (51%)	0.68 (0.36–1.32)	0.27
Any fracture	93 (57%)^4^	45 (47%)^4^	48 (72%)	0.35 (0.18–0.71)	**<0.005**
Injury mechanism: Gunshot	100 (60%)	83 (85%)	17 (25%)	15.91 (7.21–35.4)	**<0.005**
Injury mechanism: Blast	63 (38%)	14 (14%)	49 (73%)	0.06 (0.03–0.14)	**<0.005**
Injury mechanism: Other	2 (2%)	1 (1%)	1 (1%)	0.68 (0.02–26.49)	1.00

GI, gastrointestinal. Data are given in median (interquartile range) and number of patients (percentage of patients) if not otherwise stated. In cases of missing data, percentages were calculated on the total of patients without missing data. Effect sizes were calculated as the Hodges-Lehmann difference for continuous variables and odds ratios for binary variables. P-values were given by Mann-Whitney U test for continuous variables and by Fisher’s exact test for binary variables.

Any concomitant injury was defined as a fracture at a different site than the randomized wound, a urogenital injury, an injury of abdominal solid organ or GI tract, a lung injury, a penetrating injury to the thorax or trachea, or a penetrating injury to the abdomen.

^1^: Data missing for 1 patient.

^2^: Data missing for 13 patients.

^3^: Data missing for 11 patients.

^4^: Data missing for 2 patients.

^5^: Data missing for 15 patients.

^6^: Data missing for 14 patients.

^7^: Data missing for 7 patients.

^8^: Data missing for 6 patients.

^a^: Odds ratio and confidence interval not calculated due to zero-cells.

**Fig 1:**
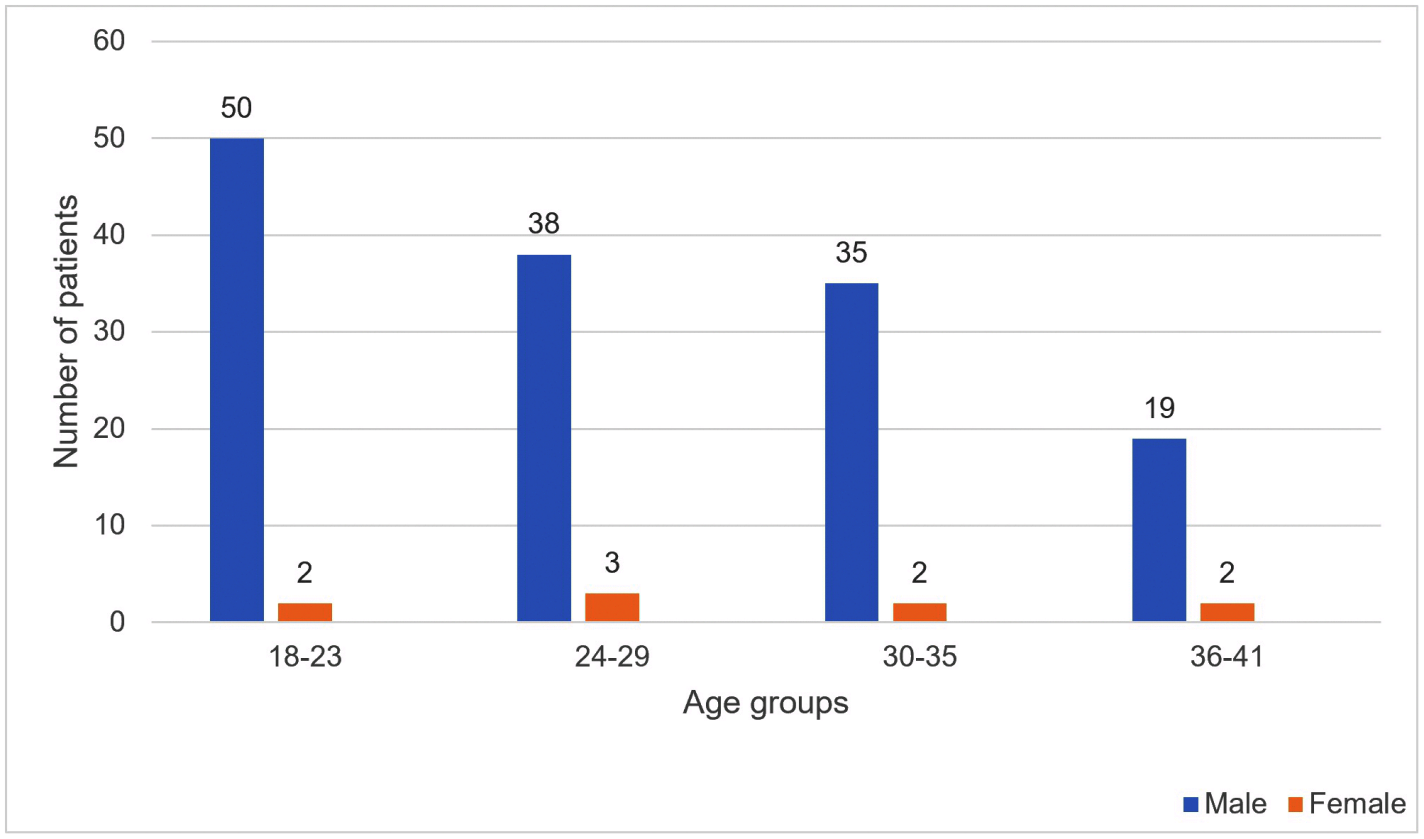
Distribution of age and sex (n = 164). One patient not included due to missing data on date of birth.

**Fig 2:**
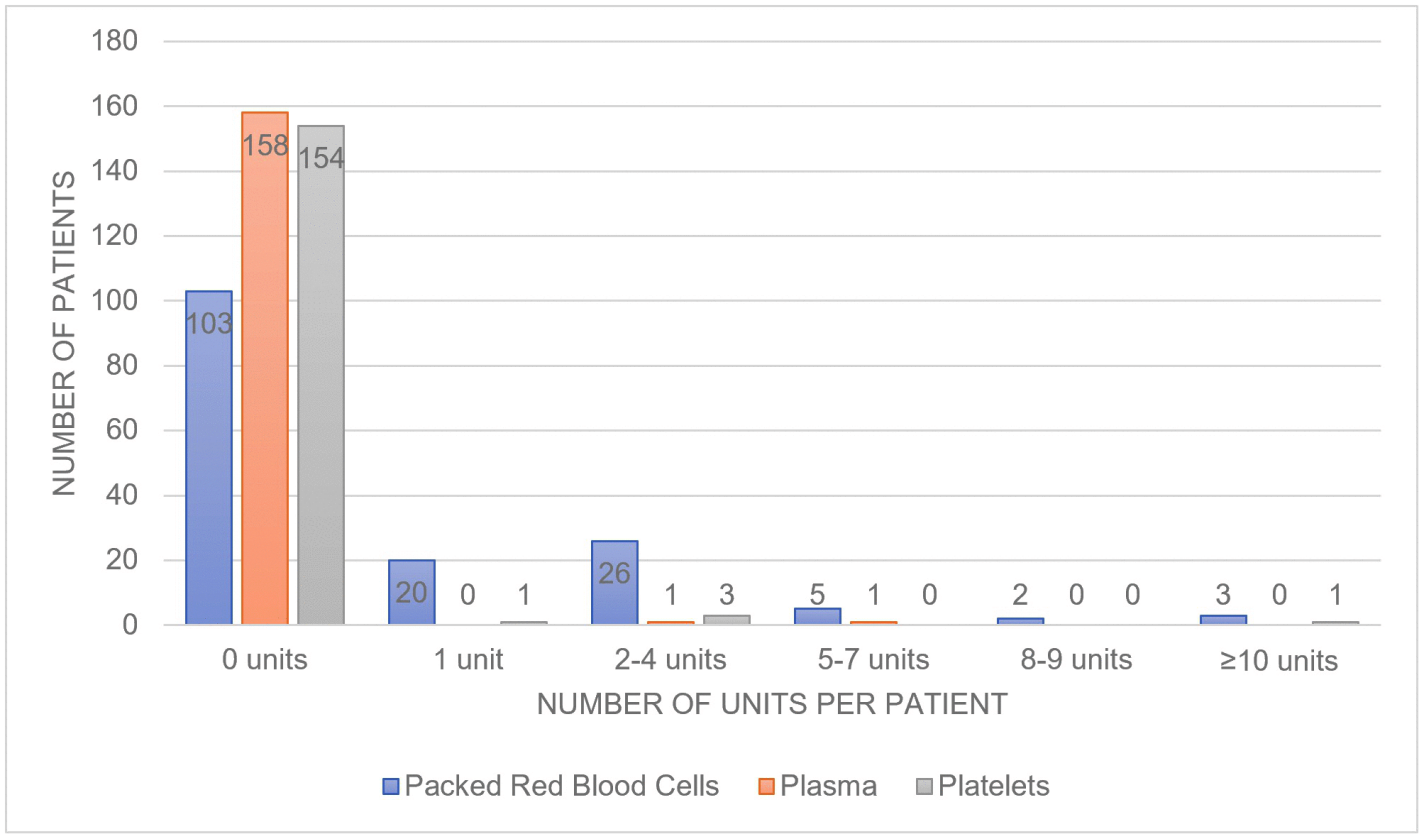
Patients by number of transfused units of blood, plasma, and platelets (n = 159 for blood, and plasma transfusions, n = 160 for platelet transfusions). Data missing for 6 patients on blood and plasma transfusions and for 5 patients on platelet transfusions.

The female patient population was about equally distributed into the age groups, whilst the male patient population age groups got smaller as age increased (Fig 1). Some 56 (35%) patients had bleeding requiring blood transfusion. The need for at least one transfusion was less common among patients arriving to Erbil than among those arriving to Ar Ramtha (21% vs 58%, OR 0.19, 95% CI 0.09–0.39). In units of packed red blood cells (PRBC), 20 patients received one unit, 26 patients received between 2 and 4 units, 5 patients received between 5 and 7 units, and 2 patients received between 8 and 9 units. Three (2%) patients received at least 10 units of PRBC (Fig 2).

The median number of days until wound closure was 5 (IQR 4–9) (Table 2). Patients treated at Ar Ramtha had a greater median number of days until closure compared with patients treated at Erbil (9 vs 4, 95% CI of difference: 2–7). After three months, the vast majority of the patients (96%) had achieved wound closure. The median length of stay was 10 days (IQR 5–37), with longer stays at Ar Ramtha (41 vs 6, 95% CI of difference: 30–40). Sepsis leading to admission to the intensive care unit affected five (3%) patients. One (1%) patient died during their stay at hospital. Among the 74 patients with an open fracture, 16 (22%) patients had a wound infection. Two (1%) patients underwent amputation, both of which had vascular injuries as well as open fractures with wound infection.

**Table 2:** Outcome characteristics.

Variable	Total (n = 165)	Erbil (n = 98)	Ar Ramtha (n = 67)	Effect size (95% CI)	P-value
Wound closure at day 5	92 (56%)	72 (73%)	20 (30%)	6.42 (3.24–13.55)	**<0.005**
Wound closure at day 14	132 (80%)	92 (94%)	40 (60%)	10.19 (3.88–27.02)	**<0.005**
Wound closure at day 30	150 (91%)	94 (96%)	56 (84%)	4.57 (1.32–16.22)	**0.01**
Wound closure at 3 months	158 (96%)	95 (97%)	63 (94%)	2.00 (0.43–10.45)	0.44
Days until wound closure	5 (4–9)^1^	4 (3–5)^2^	9 (5–18)^3^	-4 (-7–-2)	**<0.005**
Sepsis leading to admission to intensive care unit	5 (3%)	0 (0%)	5 (7%)	-^a^	**0.01**
Any amputation, number of patients	2 (1%)	0 (0%)	2 (3%)	-^a^	0.16
Bleeding leading to blood transfusion, number of patients	56 (35%)^4^	20 (21%)^5^	36 (58%)^6^	0.19 (0.09–0.39)	**<0.005**
Open fracture and wound infection, number of patients	16 (22%)^7^	6 (15%)^8^	8 (26%)^9^	0.58 (0.17–2.07)	0.39
Received prophylactic antibiotics	163 (99%)^5^	97 (100%)^5^	66 (99%)	-^a^	0.41
Discharged to home	136 (83%)^10^	88 (91%)^5^	48 (73%)^11^	3.64 (1.46–9.36)	**<0.005**
Length of stay, days	10 (5–37)	6 (5–9)	41 (26–70)	-34 (-40–-30)	**<0.005**
Death during hospital stay	1 (1%)	0 (0%)	1 (1%)	-^a^	0.41

Data are given in median (interquartile range) and number (percentage) of patients if not otherwise stated. In cases of missing data, percentages were calculated on the total of patients not missing data. Days until wound closure calculated on patients with successful wound closure (n = 158). Wound infection was defined as having purulent discharge. Effect sizes were calculated as the Hodges-Lehmann difference for continuous variables and odds ratios for binary variables. P-values were given by Mann-Whitney’s U test for continuous variables and by Fisher’s exact test for binary variables.

^1^: Data missing for 7 patients.

^2^: Data missing for 3 patients.

^3^: Data missing for 4 patients.

^4^: Data missing for 6 patients.

^5^: Data missing for 1 patient.

^6^: Data missing for 5 patients.

^7^: Data missing for 1 patient, percentage calculated on number of patients with a fracture at the site of randomized wound (an open fracture), n = 74.

^8^: n = 40.

^9^: n = 34.

^10^: Data missing for 1 patient, 1 patient dead.

^11^: 1 patient dead.

^a^: Odds ratio and confidence interval not calculated due to zero-cells.

## Discussion

Among civilians with acute conflict-related extremity injuries, open fractures were common and more than one in five patients with an open fracture developed a wound infection. The injury profile differed between the sites, with patients arriving at Ar Ramtha having more wounds, more concomitant injuries, required longer time to wound closure, and spent longer time in the hospital compared with patients treated in Erbil. Bleeding requiring transfusion was a common complication and affected one third of the patients. Mortality was low.

In the present study, 45% of the patients had an open fracture. Among the patients with an open fracture, 22% had a wound infection, operationalized in this dataset as purulent discharge. An open fracture is a significant risk factor for infection, which has been shown to be associated with poor outcomes and excess resource consumption for conflict-related injuries [21,22]. Data specifically describing infection among civilian patients with conflict-related open fractures remain limited. In one retrospective Syrian study of open long-bone fractures treated with external fixation, infection was reported in 17% of the patients [23], while a recent scoping review of peripheral extremity surgery during the Syrian conflict highlighted limited reporting of complications, poor follow-up, and incomplete data capture in the available literature [24]. Recent reports from Gaza have described high infection burdens among patients with conflict-related fractures, including suspected fracture-related infection rates of 30% and reported infection in up to 62% of fractures, although direct comparison with the present cohort is limited by differences in case definitions, injury severity, and the organization of care [25,26].

Previous research suggests that adherence to treatment guidelines can be difficult in conflict settings [27]. The observed infection rate in our study may partly reflect protocolized wound management consistent with guidelines, including avoidance of primary closure, debridement according to war-surgery principles, and near-universal antibiotic prophylaxis (99%) [10]. However, antibiotics should be interpreted as an adjunct to adequate surgical management rather than as an isolated explanation for infection prevention. Our findings are compatible with the hypothesis that high adherence to protocolized wound care, including antibiotic prophylaxis, may contribute to infection prevention in civilian conflict-related open fractures. In this context, the present prospectively collected cohort adds specific infection data for open fractures from a civilian conflict-injury population with standardized wound-management variables, including closure strategy and antibiotic prophylaxis.

Site-stratified analyses showed that patients treated at Ar Ramtha had a higher burden of wounds, concomitant injuries, complications, and blast-related injuries compared with patients treated at Erbil. These differences were accompanied by longer times to wound closure and longer hospital stays, suggesting that the two sites captured distinct civilian conflict-injury case mixes with different surgical and resource requirements. Such site-level differences are relevant for public health planning in conflict settings, including referral pathways, operative capacity, wound-care resources, rehabilitation needs, and hospital bed availability. Our results are consistent with previous literature showing heterogeneity in civilian injury patterns across sites within the same conflict [28–30]. However, comparisons between sites can be limited by heterogeneous retrospective data. In the present study, injury mechanism, wound closure, complications, and length of stay were collected prospectively within the same trial. Therefore, the study provides standardized data on both injury patterns and outcome measurements. Nevertheless, while the randomized trial framework standardized measurement across sites, the observed differences may still partly reflect unmeasured variation in local treatment guidelines and surgical decision-making that was not fully specified or captured by the study protocol.

We found that 35% of the patients had received at least one unit of PRBC. Bleeding is a common complication and a significant cause of mortality among patients with conflict-related injuries [31]. A 2021 study on predicting surgical resource consumption at civilian hospitals in resource-limited conflict settings found a blood transfusion rate of 20% [32], whilst a recent study on a French military mission treating civilians injured in Gaza found a blood transfusion rate of 14% [33]. In comparison, our patient population had a high rate of blood transfusions. One possible systems-level explanation for this may be a difference in the availability of blood; our study hospitals were supplied via central regional blood banks, which may have facilitated easier access to PRBCs. However, this explanation remains uncertain, as whilst the 2021 study had no available data on blood product availability, the French study in Gaza noted a regular supply of blood products [32,33]. It is also possible that since our study did not include patients injured in mass casualty events, blood transfusions may have been done more liberally; mass casualty events can lead to more strict rationing of the local supply of blood products [34]. Finally, it is also possible that our results reflect our studied population; civilians with acute conflict-related extremity wounds requiring surgical care. Because specific transfusion indications and thresholds were not compared across settings, these interpretations should remain cautious.

Mortality was low at 1%. Studies comparing the mortality at civilian trauma hospitals during conflicts in resource-limited settings to civilian trauma hospitals in non-conflict, resource-rich settings have found the mortality in resource-limited conflict settings to be markedly lower [5,35]. There are several factors that might contribute to these differences. A study on the civilian pre-hospital care during the Battle of Mosul, Iraq demonstrates several obstacles to providing effective care to injured civilians near the front, including disturbances by military personnel, a lack of ambulance drivers, and the risk of harm to first responders [36]. Thus, conflict-related civilian trauma research probably suffers from survivorship bias where the most injured patients die before reaching the hospital due to inadequate pre-hospital care, which our results corroborate. Due to lack of neurosurgical and burns services at the hospitals there were no concomitant injuries of the CNS or severe burns. Previous studies on civilian casualties of armed conflict have shown that CNS injuries carry a severely increased risk of mortality as compared to non-CNS injuries [4]. Furthermore, studies in non-conflict settings on combined burn and trauma injuries have shown that these injuries carry severely increased mortality as compared to trauma injuries without burns [37,38]. However, previous research on civilian patients including those suffering head and neck injuries as well as burn injuries during the same conflict and in similar hospital settings as our study found similar results as to mortality [5,39].

### Limitations

Limitations to this study include the studied population being patients reaching civilian trauma hospitals. It is likely that this introduced survivorship bias, since the hospitals received patients stable enough and injured in such a manner to survive the lack of appropriate pre-hospital care. This risk of bias is probably exacerbated by patients with CNS injuries and severe burns being referred to other hospitals with neurosurgical and burns services, as these injuries carry a severely increased risk of death [4,37,38]. However, it should be noted that given the issues with collecting data close to point of injury in conflict settings due to violent and chaotic conditions, studying patients reaching hospital together with an awareness of this bias is an accessible method of indirectly studying gaps in pre-hospital care [5,36]. Furthermore, the studied population is relevant to healthcare providers, as it is a population able to receive care. Another limitation to this study is including only patients with wounds not suitable for primary closure. However, current gold standard treatment recommends primary closure of conflict-related injuries only under very rare circumstances [10]. Specific transfusion indications were not available per site, which limits the interpretation of transfusion rates. Lastly, exclusion of patients injured in mass casualty events and of patients treated when a research nurse was unavailable may have limited generalizability and introduced selection bias. The study was conducted in an active conflict context; although data collection was prospective and standardized, conflict-related disruption may still explain part of the observed missingness.

## Conclusions

Among civilians treated for acute conflict-related extremity injuries, fractures were common, open fractures were frequent, and bleeding requiring transfusion affected a substantial proportion of patients. Infection among patients with open fractures was comparatively uncommon, possibly reflecting protocolized wound management, although differences in case mix and organization of care should be considered. Low in-hospital mortality likely reflects, at least in part, the selection of patients who survived long enough to reach hospital. These prospectively collected data provide a standardized description of an underreported civilian conflict-injured population and may inform planning for surgical, transfusion, and wound-care capacity in similar settings.

## Supporting information

S1 Checklist Inclusivity in global research. (DOCX)
